# Mouse Tissue‐Resident Peritoneal Macrophages in Homeostasis, Repair, Infection, and Tumor Metastasis

**DOI:** 10.1002/advs.202206617

**Published:** 2023-01-19

**Authors:** Carlos Ardavín, Natalia Alvarez‐Ladrón, Margarita Ferriz, Alejandra Gutiérrez‐González, Adrián Vega‐Pérez

**Affiliations:** ^1^ Departamento de Inmunología y Oncología Centro Nacional de Biotecnología/CSIC Darwin 3 Madrid 28049 Spain; ^2^ Present address: Sandra and Edward Meyer Cancer Center Weill Cornell Medicine 1300 York Avenue New York NY 10065 USA

**Keywords:** macrophages, monocyte‐derived macrophages, peritoneal cavity, peritoneal injury, peritoneal metastasis, peritoneal sepsis, tissue‐resident macrophages

## Abstract

Large peritoneal macrophages (LPMs) are long‐lived, tissue‐resident macrophages, formed during embryonic life, developmentally and functionally confined to the peritoneal cavity. LPMs provide the first line of defense against life‐threatening pathologies of the peritoneal cavity, such as abdominal sepsis, peritoneal metastatic tumor growth, or peritoneal injuries caused by trauma, or abdominal surgery. Apart from their primary phagocytic function, reminiscent of primitive defense mechanisms sustained by coelomocytes in the coelomic cavity of invertebrates, LPMs fulfill an essential homeostatic function by achieving an efficient clearance of apoptotic, that is crucial for the maintenance of self‐tolerance. Research performed over the last few years, in mice, has unveiled the mechanisms by which LPMs fulfill a crucial role in repairing peritoneal injuries and controlling microbial and parasitic infections, reflecting that the GATA6‐driven LPM transcriptional program can be modulated by extracellular signals associated with pathological conditions. In contrast, recent experimental evidence supports that peritoneal tumors can subvert LPM metabolism and function, leading to the acquisition of a tumor‐promoting potential. The remarkable functional plasticity of LPMs can be nevertheless exploited to revert tumor‐induced LPM protumor potential, providing the basis for the development of novel immunotherapeutic approaches against peritoneal tumor metastasis based on macrophage reprogramming.

## Graphical abstract

Research performed over the last few years that has widened the knowledge on resident peritoneal macrophages by unveiling the mechanisms by which these cells fulfill a crucial role in repairing peritoneal injuries, form mesothelium‐bound immune aggregates to control microbial infections, and promote peritoneal metastatic tumor growth.

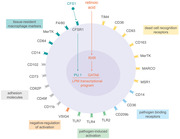

## 1 Introduction

The peritoneal cavity, as well as the pleural and pericardial cavities, are generated, from the embryonic coelome, a cavity resulting from the formation of the embryonic body wall, comprising the parietal plate mesoderm and the ectoderm, and the gut wall, comprising the visceral plate mesoderm and the endoderm.^[^ ^1^ ^]^ The process by which the embryonic coelome is formed has been conserved from the primitive superphyla Protostomia and Deuterostomia, so that invertebrates of the phyla Annelida, Mollusca, Echinodermata, and Tunicata possess a coelomic cavity anatomically and developmentally equivalent to the embryonic coelome,^[^ ^2^ ^]^ that generates the peritoneal, pleural, and pericardial cavities during the embryonic development of mammals.

The peritoneal cavity is covered by the peritoneum, the largest serous membrane of the body, with a surface area comparable to that of the skin, composed by the mesothelium, an epithelium of mesodermal origin, a basal membrane, and a submesothelial connective tissue.^[^ ^3^ ^]^

The parietal peritoneum lines the inner surface of the abdominal wall, whereas the visceral peritoneum integrates with the serosal layers of intra‐abdominal organs. A double fold of the peritoneum forms the mesentery, that connects abdominal digestive organs to the abdominal wall, and serves as a conduit for vessels, nerves, and lymphatics. A small volume of peritoneal fluid secreted by mesothelial cells serves as a lubricant in the peritoneal cavity, and prevents mechanical friction between abdominal organs. In mice, total peritoneal fluid volume was estimated in two recent reports to be around 50–100 µL in the steady state,^[^ ^4^, ^5^ ^]^ and was claimed to differ between males and females (≈20 µL vs ≈100 µL) and, in the latter, to change during the estrous cycle.^[^ ^6^ ^]^ Drainage of the peritoneal fluid into the lymphatic system allows peritoneal fluid recirculation,^[^ ^7^ ^]^ and is achieved through openings in the mesothelium, called stomata, that are mainly located in the diaphram and omentum.^[^ ^3^ ^]^ The omentum is a visceral adipose tissue that develops by overgrowth of the mesentery and harbors a specialized vascular system and an organized lymphoid tissue, claimed to play an important role in defense against peritoneal infection.^[^ ^8^ ^]^ Peritoneal fluid draining through the diaphragm collects into the subperitoneal lymphatic lacunae to reach the diaphragm collecting lymphatics, that drain into the mediastinal lymph nodes, whereas peritoneal fluid draining through the omentum collects in the omental lymphatics, that in turn collect into the intestinal lymphatic trunk that connects to the thoracic duct through the cisterna chyli.^[^ ^3^ ^]^ Drainage of the peritoneal cavity allows control of peritoneal homeostasis and leukocyte recirculation, but increases the risk of pathogen and metastatic tumor cell dissemination.

The peritoneal cavity is exposed to two major pathologies, infection and tumor metastasis, generally associated with a high mortality, due to the easy spreading of pathogens or tumor cells throughout intra‐abdominal organs, and to the anatomical features of the peritoneal cavity that greatly hinders the development of efficient treatments against these diseases. Despite the fact that the peritoneal cavity is a confined space, not readily exposed to invading pathogens such as those penetrating the skin, the lungs or the gut, peritoneal infections can arise due to the loss of intestinal wall integrity (caused by ulcers, strangulation of hernias, appendicitis, or tumor growth), liver cirrhosis, accidental abdominal injuries, abdominal surgery, or peritoneal dialysis. The peritoneal cavity is also exposed to injuries in the parietal or visceral peritoneum caused by trauma, infection, or abdominal surgery, which can lead to peritoneal adhesions. Additional pathologies of the peritoneal cavity include—peritoneal endometriosis, involving the formation of ectopic vascularized endometrial tissue in the peritoneum associated with chronic inflammation—peritoneal autoimmune serositis, a chronic inflammation of the peritoneum caused by autoimmune diseases, such as Crohn's disease and—post‐surgical peritoneal adhesions.^[^ ^3^, ^9^, ^10^ ^]^

Immune defense against peritoneal infection and tumor metastasis relies on a first line of local defense supported by resident peritoneal immune cells, present in the peritoneal cavity in the steady state, with innate immunity sensing and responding properties. The second line of immune defense in the peritoneal cavity is provided by functional units of lymphoid tissue, associated to adipose tissue located in the omentum, mesentery or gonadal fat, called fat‐associated lymphoid clusters (FALCs), or milky spots for omental FALCs.^[^ ^8^ ^]^ FALCs harbor an structural organization similar to that found in secondary lymphoid organs, including a reticular cell‐based stroma, B and T cell compartments, and specialized blood and lymphatic vessels, allowing leukocyte migration to and from the peritoneal cavity.^[^ ^8^ ^]^

Resident peritoneal immune cells include tissue‐resident peritoneal macrophages, generally named large peritoneal macrophages (LPMs) and B1 cells. Recent experimental evidence has unveiled that, apart from their primary phagocytic function, LPMs fulfill different homeostatic, repair and immunological defense functions, that reflect a previously unexpected functional plasticity.^[^ ^11^ ^]^ Peritoneal B1 cells are considered as innate‐like B cells, that constitutively produce natural IgM, providing local immune protection against a wide variety of pathogens. In addition, B1 cells actively produce IgM in response to viruses, bacteria, fungi, and parasites.^[^ ^12^ ^]^ The first line of immunity in the peritoneal cavity in mammals, relying on phagocytic and antibody‐mediated defense mechanisms supported by LPMs and B1 cells, is reminiscent of the primitive defense mechanisms sustained by different populations of coelomocytes present in the coelomic cavity of invertebrates.^[^ ^13^, ^14^, ^15^ ^]^ Immune defense strategies in coelomic cavities have been therefore highly conserved throughout evolution from invertebrates to higher vertebrates.

In this review, we discuss recent evidence that has widened our knowledge on the biology of LPMs by describing the mechanisms of resident embryonic LPM replacement by resident bone marrow monocyte‐derived LPMs (moLPMs), that result in phenotypic and functional LPM sexual dimorphism, and unveiling how LPMs, free in a fluidic environment in the steady state, perform repair and immune defense functions, by forming thrombus‐like structures in response to peritoneal injury, and mesothelium‐bound dynamic LPM aggregates after bacterial infection. Moreover, recent experimental evidence support that peritoneal tumors can subvert LPM metabolism, leading to the acquisition of tumor‐promoting functions that, nevertheless, might be reverted by experimental strategies blocking tumor‐induced subversion of LPM function, that could be the basis for the development of novel immunotherapeutic approaches against peritoneal tumor metastasis based on macrophage reprogramming.

## 2 Large Peritoneal Macrophage Identity

LPMs are long‐lived, tissue‐resident macrophages formed during embryonic life, developmentally and functionally confined to the peritoneal cavity, in contrast to other peritoneal immune cell populations that are recruited to the peritoneal cavity and recirculate to other locations in the steady state, and under pathological conditions. Those include, in the steady state, B1 cells, that together with LPMs constitute the vast majority of cells harvested by peritoneal lavage, and a low percentage of monocyte‐derived SPMs (for small peritoneal macrophages), B2 cells, T cells, NK cells, innate lymphoid cells, and mast cells.^[^ ^11^ ^]^ As discussed in depth in this review, research performed along the last years has established that LPMs not only fulfill peritoneal homeostatic functions, but are also involved in repair of tissue damage caused by inflammation and infection, and defense against microbial infection. Moreover, LPMs contribute to most peritoneal pathologies, particularly to peritoneal tumor metastasis, but also to peritoneal endometriosis, autoimmune serositis, and post‐operative adhesions.

**Figure 1:**
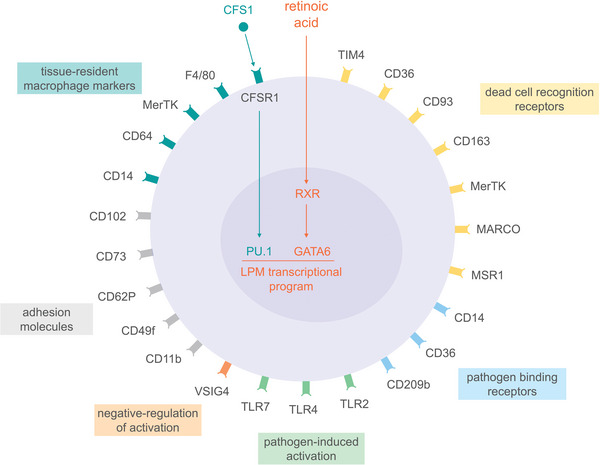
Schematic representation of cell surface receptors expressed by embryonic LPMs. The LPM transcriptional program driven by CFS1 and retinoic acid, through the transcription factors PU.1 and GATA6, controls the expression of cell surface molecules characteristic of embryonic LPMs, such as markers of tissue‐resident macrophages, adhesion molecules, and receptors involved in recognition of dead cells, pathogen binding, pathogen‐induced activation, and negative‐regulation of LPM activation. Of note, only the most representative cell surface molecules expressed by embryonic LPMs have been included in this figure.

Resident embryonic LPMs are CD11b^+^ F4/80^hi^ MHC‐II^−^ cells expressing a series of markers characterizing tissue‐resident macrophages, such as CD14, CD64, and MerTK.^[^ ^16^, ^17^ ^]^ Besides, tissue‐resident macrophages located in the serous cavities of the body, that comprise LPMs and tissue resident macrophages present in the pleural and pericardial cavities, appear to share the expression of the transcription factor GATA6, the scavenger receptor Tim4, and the M‐CSF receptor CFSR1.^[^ ^11^, ^18^ ^]^ In addition, resident embryonic LPMs are characterized by the expression of a number of cell surface receptors reflecting LPM homeostatic, repair, regulatory and defense functions, including molecules involved in LPM adhesion and localization, such as ICAM‐2 (CD102), CD11b, CD49f, CD73, and CD62P,^[^ ^19^ ^]^ recognition and removal dead cells, such as CD36, CD93, CD163, Tim4, MerTK, MARCO, and MSR1,^[^ ^4^, ^16^, ^20^, ^21^, ^22^ ^]^ negative regulation of macrophage activation, ensuring non‐inflammatory clearance of apoptotic cells, such as V‐set immunoglobulin domain‐containing 4 (VSIG4),^[^ ^23^ ^]^ pathogen binding, such as CD14, CD36, and SIGN‐R1 (CD209b)^[^ ^11^, ^24^ ^]^ and response to pathogens, such as TLR4 and TLR7.^[^ ^25^, ^26^ ^]^ The most representative cell surface molecules expressed by embyonic LPMs are summarized in **Figure** **1**.

LPMs belong to the family of tissue‐resident macrophages, that share the expression of core lineage related genes determined during embryonic life, but acquire tissue‐specific transcriptional and functional features established upon exposure to tissue specific microenvironmental signals, through the expression of tissue‐specific transcription factors.^[^ ^16^, ^27^ ^]^ In this regard, the transcription factor GATA6 is essential for LPM‐specific gene expression, proliferation, and survival of LPMs.^[^ ^19^, ^28^, ^29^ ^]^ Consequently, homeostatic, repair, and defense LPM functions were compromised in mice deficient in GATA6 in myeloid cells.^[^ ^5^, ^19^, ^30^ ^]^ GATA6 expression is maintained in a non‐cell autonomous manner^[^ ^27^, ^31^ ^]^ and was proposed, based on in vitro experiments, to be activated by the vitamin A metabolite retinoic acid, through retinoic acid nuclear receptors.^[^ ^19^ ^]^ GATA6 expression would be thus modulated by the local availability of retinoic acid, supporting the concept that the GATA6‐induced transcriptional program of LPMs is reversible,^[^ ^17^ ^]^ which would be the basis for the functional plasticity of LPMs, that enables LPMs to switch from homeostatic to repair or immune defense functions when needed. In this regard, LPMs transferred into the alveolar space downregulated GATA6 and acquired an alveolar macrophage transcriptional profile.^[^ ^27^ ^]^ Retinoic acid activating GATA6 in LPMs was claimed to be produced by omental and peritoneal stromal cells.^[^ ^19^ ^]^ In line with these observations, expression by mesothelial and fibroblastic stromal cells of the Wilms' tumor 1 (WT1) transcription factor, that drive the expression of two rate‐limiting enzymes controlling in retinol metabolism, RALDH1 and RALDH2,^[^ ^32^ ^]^ was claimed to control GATA6 expression in LPMs and in GATA6^+^ resident macrophages located in the pleural and pericardial cavities, since depletion of WT1^+^ cells in resulted in a profound reduction in these macrophage subsets, paralleled by a concomitant dimishment of *Raldh1* and *Raldh2* transcripts,^[^ ^18^ ^]^ further supporting the role of retinoic acid in sustaining GATA6 expression, that nevertheless remains to be formally demonstrated. The fact that in GATA6‐deficient mice CD11b^+^ macrophages accumulated in omental milky spots, while LPMs were reduced in the peritoneal cavity,^[^ ^19^ ^]^ supports the hypothesis that retinoid acid secreted by stromal cells in the omentum maintains the GATA6‐driven transcriptional program in LPMs, and would imply that LPMs continuously recirculate through the omentum, but this remains to be formally demonstrated.

Retinoic acid is a ligand of retinoid X receptors (RXRs), which are members of the nuclear receptor superfamily of ligand‐dependent transcription factors, that control lipid and glucose metabolism, and play key roles in inflammatory and autoimmune disorders.^[^ ^33^ ^]^ Interestingly, mice deficient in RXRs *α* and *β* displayed a profound defect in neonatal LPM expansion, and a reduced survival of adult LPMs, due to lipid accumulation resulting in apoptosis, revealing that RXRs contribute to the expansion and maintenance of LPMs.^[^ ^34^ ^]^ ATAC‐seq analyses revealed that the *Gata6* locus displayed reduced chromatin accessibility in RXR‐deficient LPMs, that correlated with a lower *Gata6* gene expression, supporting that RXRs regulate the GATA6‐dependent LPM transcriptional program.

Macrophage colony stimulating factor (M‐CSF or CFS1) control commitment to the macrophage lineage, and therefore LPM differentiation is dependent on CFS1, as demonstrated in osteopetrotic (*Csf1 ^op/op^*) mice that harbor a mutation in the *Cfs1* gene, leading to a defective LPM development.^[^ ^35^ ^]^ Moreover, based on in vitro assays, mesothelial cells were reported to secrete CSF1 that sustained LPM proliferation in mesothelial cell‐LPM co‐cultures; transwell assays revealed that LPM proliferation was significantly reduced when mesothelial‐LPM interactions were prevented, suggesting that cell‐to‐cell contact contributed to LPM proliferation.^[^ ^36^ ^]^ The concept that mesothelial‐derived CSF1 is required for LPM maintenance is further supported by a recent report showing that LPMs were highly reduced in mice in which WT1^+^ cells were deficient in CFS1.^[^ ^37^ ^]^ Whether mesothelial cell‐derived CSF1 contributes to steady state LPM self‐renewal and/or to LPM proliferation during inflammation remains to be explored.

## 3 Large Peritoneal Macrophage Origin and Replacement in Homeostasis

**Figure 2:**
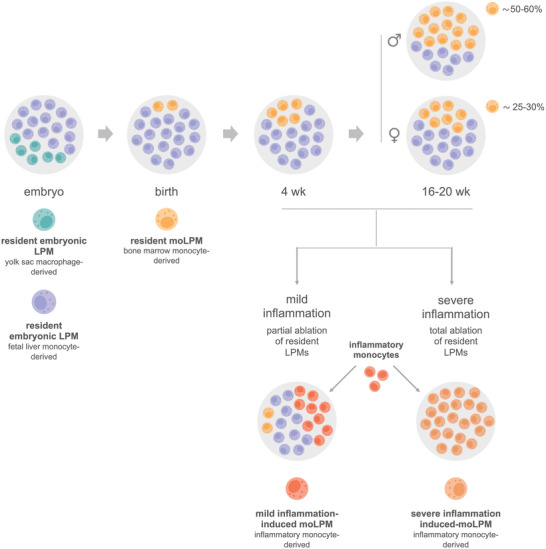
Integrated model of the origin and replacement of LPMs in steady state and inflammation. LPMs differentiate during embryonic life and maintain themselves by in situ self‐renewal during adult life. Resident embryonic LPMs were claimed to derive either from a dual contribution from yolk sac macrophages and fetal liver monocytes, or exclusively from fetal liver monocytes. Embryonic LPMs are gradually, yet partially, replaced from the late stages of embryonic development by resident moLPMs, that acquire a resident embryonic LPM identity, but retained some transcriptional and functional characteristics related to their origin. Changes in the peritoneal microenvironment, that arise upon sexual maturity, leads to a sexually dimorphic replacement of embryonic LPMs by resident moLPMs, the replacement rate being higher in males than in females. Inflammatory reactions in the peritoneal cavity can lead to resident LPM cell death leading to a reduction in the number of resident LPMs, whose extent correlates with the severity of inflammation. Recovery of the original LPM pool occurs by proliferation of the remaining resident LPMs and replacement by ii‐moLPMs. ii‐moLPMs formed after mild inflammation co‐exist long‐term with remaining resident LPMs, but do not acquire a resident LPM phenotype, due to competition with resident LPMs and alterations in peritoneal environment. In contrast, severe inflammation can lead to the total ablation of resident LPMs, which are ultimately replaced by ii‐moLPMs, that acquire a resident LPM identity, but maintained transcriptionally and functionally divergent features, determined by their origin, peritoneal inflammation, and time‐of‐residency.

LPMs differentiate during embryonic life and maintain themselves by in situ self‐renewal during adult life. In the steady state, embryonic LPMs are gradually, yet partially, replaced from the late stages of embryonic development by resident bone marrow moLPMs that acquire a resident embryonic LPM identity, but retained some transcriptional and functional characteristics related to their origin.^[^ ^38^, ^39^ ^]^ The origin of embryonic LPMs remains controversial since they were reported to derive either from a dual contribution from yolk sac macrophages and fetal liver monocytes,^[^ ^40^ ^]^ or exclusively from fetal liver monocytes.^[^ ^41^ ^]^ An integrated model of the origin and replacement of LPMs is shown in **Figure** **2**. The replacement of embryonic for bone marrow monocyte‐derived tissue‐resident macrophages, in the steady state, has been described for all tissue‐resident macrophage populations, except microglia, Langerhans cells and Kupffer cells, as reported by Dr. F. Ginhoux's lab, using fate‐mapping models based on the expression of the *Ms4a3* gene, specifically expressed by granulocyte‐monocyte progenitors.^[^ ^42^ ^]^ The degree of replacement by bone marrow monocyte‐derived macrophages appears to be essentially dictated by niche access and availability.^[^ ^43^ ^]^ None of the tissue‐resident macrophage populations exhibit a total replacement by bone marrow monocyte‐derived macrophages, suggesting that an equilibrium is reached in each organ between bone marrow monocyte recruitment, and proliferation and survival of embryonic and bone marrow monocyte‐derived resident macrophages.^[^ ^42^ ^]^

Therefore, during adult life the resident LPM pool is maintained, in the steady state, by a combination of self‐renewal of resident embryonic LPMs and differentiation and self‐renewal of resident moLPMs. Consequently, in this manuscript, unless otherwise indicated, the term LPMs refers to the adult LPM population which, in the steady state, comprises resident embryonic LPMs and resident moLPMs. Interestingly, after sexual maturity, the rate of embryonic LPM replacement is higher in males, whose LPMs display a higher proliferative activity, as demonstrated by genetic fate‐mapping analyses from Dr. F. Ginhoux's and Dr. S. Jenkins' labs, that nevertheless reported differences in the rates of replacement.^[^ ^39^, ^42^ ^]^ Ginhoux and colleagues^[^ ^42^ ^]^ found a higher proportion of resident moLPMs in males at 8 weeks and 20 weeks of age (≈25% vs 10% and ≈50% vs 25%, respectively). In contrast, Jenkins and colleagues^[^ ^39^ ^]^ reported that ≈30% resident moLPMs were detected both in males and females at 4 weeks, whereas at 16 weeks, males harbored a higher proportion of resident moLPMs (≈60% vs 30%). Sexually dimorphic replacement by resident moLPMs was proposed to be controlled by changes in the peritoneal microenvironment that arise upon sexual maturation, independently of estrogen levels and peritoneal adiposity,^[^ ^39^ ^]^ leading to divergence in the heterogeneity of the LPM population. Sex‐associated divergence in the heterogeneity within the LPM population, as well as sex differences in the peritoneal microenvironment, determine significant transcriptional and functional differences between the LPM population in male and female mice, although RNA‐seq analyses at the single‐cell level revealed equivalent cluster identities in male and female LPMs. RNA‐seq analyses, at population‐level, of 10‐ to 12‐week‐old male and female mice LPMs indicated that 486 mRNA transcripts were differentially expressed (>1.5‐fold) between female and male LPMs. The 148 mRNA transcripts more highly expressed in female LPMs, at the population level, comprised genes associated with lipid uptake and transport, such as *Apoe*, *Apoc1*, S*aa2*, and *Saa3*, as well as, genes associated with immune defense. The latter included *Timd4*, *Cxcl13*, *Tgfb2*, the complement component genes *C1qa*, *C3*, and *C4b* and the C‐type lectin receptor genes *Cd209a*, *Cd209b*, and *Clec4g*.^[^ ^39^ ^]^ In contrast, in males, the genes more highly expressed by LPMs were associated with proliferation and cell cycle‐related processes, such as *Cdk1*, E2f2, and *Mki67*.

Interestingly, female mice were more resistant to acute peritonitis induced by group B streptococci^[^ ^44^ ^]^ or by *Streptococcus pneumoniae* infection.^[^ ^39^ ^]^ Since CD209 (SIGN‐R1) is critical for survival after infection by *S. pneumoniae* infection by promoting efficient bacterial phagocytosis and clearance,^[^ ^24^ ^]^ the sex‐dependent resistance to bacterial peritonitis has been claimed to be due to the higher expression by female LPMs of CD209 and, additionally, of complement components and of the B1 cell‐recruiting chemokine CXCL13.^[^ ^39^ ^]^ In this regard, the higher resistance of women and infants to blood‐borne infections was claimed to correlate with an enhanced CXCL13‐dependent production by B1 cells of natural antibodies.

## 4 Large Peritoneal Macrophage Replacement Induced by Inflammation

Inflammatory reactions in the peritoneal cavity induced by sterile inflammatory stimuli,^[^ ^5^, ^39^, ^42^, ^45^, ^46^ ^]^ abdominal surgery,^[^ ^39^ ^]^ or bacterial infection^[^ ^47^ ^]^ were reported to cause LPM cell death leading to a reduction in the number of resident LPMs (including resident embryonic LPMs and resident moLPMs), whose extent correlates with the severity of inflammation.^[^ ^42^, ^46^ ^]^ Recovery of the original LPM pool occurs by proliferation of the remaining resident LPMs^[^ ^45^ ^]^ and replacement by LPMs derived from inflammatory monocytes (hereafter ii‐moLPMs for inflammation‐induced moLPMs) as demonstrated using different experimental strategies, based on fate‐mapping models,^[^ ^42^ ^]^ tissue‐protected bone marrow chimeric mice and adoptive transfer experiments.^[^ ^39^, ^46^ ^]^

Using an experimental model based on the induction of mild inflammation, caused by low‐dose zymosan (10 µg per mouse), or severe inflammation caused by high‐dose zymosan (1000 µg per mouse), and adoptive transfer experiments to track ii‐moLPMs and assess how the inflammatory environment controls their differentiation, Jenkins and colleagues proposed that the degree of replacement of resident LPMs by ii‐moLPMs, and the extent to which the later acquire the identity and function of resident LPMs is determined by the severity of the inflammatory process and the magnitude of LPM death^[^ ^46^ ^]^ (Figure 2).

ii‐moLPMs formed after mild inflammation co‐existed long‐term with remaining resident LPMs, but competition with resident LPMs and alterations in peritoneal environment retained them in an aberrant state of activation, and blocked the acquisition of a resident LPM phenotype. In contrast, severe inflammation can lead to the total ablation of resident LPMs, which are ultimately replaced by ii‐moLPMs, that acquired a resident LPM identity, but maintained transcriptionally and functionally divergent features, determined by their origin, peritoneal inflammation, and time‐of‐residency.^[^ ^46^ ^]^ The phenotype of ii‐moLPMs was proposed to comprise intrinsic markers determined by their origin, such as CD62L and Semaphorin 4a, markers whose expression is controlled by competition with resident LPMs but is reprogrammed with time, such as GATA6, MHCII, and CCR5, and markers related to time‐of‐residency, independent of competition with resident LPMs, such as Tim4, CD209b, and VSIG4. A significant proportion of genes differentially expressed by resident LPMs and ii‐moLPMs appear to be controlled by differences in retinoic acid signaling, either directly or in a GATA6 dependent manner.^[^ ^46^ ^]^

ii‐moLPMs exhibit a higher proliferative activity than resident LPMs,^[^ ^38^, ^46^ ^]^ that was suggested to correlate with differences in the enhanced ability of the former to proliferate in response to CSF1 produced by mesothelial cells.^[^ ^36^ ^]^ In addition, ii‐moLPMs displayed a lower ability to phagocytose bacteria and uptake dying cells, and failed to produce CXCL13.^[^ ^46^ ^]^ While the number of peritoneal B1 cells increase with age in homeostasis, peritoneal inflammation led to a defective accumulation of B1 cells^[^ ^46^ ^]^ since, as pointed out above, CXCL13 production by LPMs control B1 cell homing to the peritoneal cavity.^[^ ^48^ ^]^ Therefore, the fact that developmental and functional heterogeneity of the LPM population depends on sex and age has important implications when addressing the role of LPMs in repair, defense, and implication in peritoneal tumor metastasis, that need to be taken into account in futures studies.

It is important to note that monocytes recruited to the peritoneal cavity during inflammatory reactions, related to non‐infectious peritoneal damage, infection, or metastatic tumor growth, can potentially differentiate into monocyte‐derived cells that fulfill specific repair, defense, or tumor‐promoting functions, but might not acquire phenotypic or functional LPM characteristics, and thus should not be considered ii‐moLPMs. However, defining the identity of cells differentiated from monocytes recruited to the inflamed peritoneum can be controversial since in most reports focusing on the functional relevance of peritoneal monocyte‐derived cells, the time‐of‐persistency, and/or acquisition of LPMs features by these monocyte‐derived cells was not addressed and, inversely, in reports on resident LPM replacement during inflammation, the function of ii‐moLPMs was not explored in‐depth.

In line with the hypothesis that competition for a particular physical niche, defined by cellular and molecular microenvironmental factors, determines the contribution of monocytes to tissue resident macrophages,^[^ ^43^ ^]^ the existence of a biochemical niche for peritoneal resident macrophages was proposed.^[^ ^46^ ^]^ Accordingly, competition for signals and cell‐to‐cell interactions controlling survival, proliferation and function of LPMs would control the balance between resident LPMs and ii‐moLPMs, as well as, the acquisition of mature resident LPM identity by ii‐moLPMs.

## 5 Role of Large Peritoneal Macrophages in Peritoneal Homeostasis

LPMs fulfill an essential role in the clearance of apoptotic cells at steady state, a hallmark of tissue‐resident macrophages that is crucial for the maintenance of self‐tolerance,^[^ ^49^ ^]^ through the expression of specific scavenger receptors including CD36, CD93, CD163 Tim4, and MerTK.^[^ ^16^, ^20^, ^21^, ^22^ ^]^ Interestingly, efficient internalization of apoptotic cells by LPMs was proposed to rely on initial binding to Tim4 of phosphatidylserine exposed by apoptotic cells, followed by MerTK‐mediated engulfment.^[^ ^20^ ^]^ LPMs are programmed by the peritoneal microenvironment to efficiently scavenge apoptotic cells, avoiding inflammation driven by TLR‐mediated by self‐derived nucleic acid recognition, while maintaining the ability to respond to infection.^[^ ^25^ ^]^ The transcription factors Kruppel‐like factors 2 and 4 were claimed to drive LPM programming for immunologically silent clearance of apoptotic cells, by controlling the expression of apoptotic cell recognition receptor genes, such as *Timd4*, *Marco*, *and Olr1*, and genes acting as negative regulators of TLR signaling, such as *Hes1*, *Socs3*, *Pdlim2*, *Ptpn6*, and *Tnfaip3*, resulting in an increased threshold of activation.^[^ ^25^ ^]^ In line with these observations, LPMs express VSIG4, a B7 family‐related receptor, reported to downregulate macrophage activation, through PDK2‐mediated reprogramming of mitochondrial pyruvate oxidation and ROS production.^[^ ^23^ ^]^

LPMs play a pivotal role in maintaining peritoneal B1 cell homeostasis. Indeed, peritoneal B1 cells, that constitutively produce natural IgM, providing a local first line of defense against a wide variety of pathogens,^[^ ^12^ ^]^ depend on the chemokine CXCL13, produced by LPMs and stromal cells, for their recruitment from the circulation and homing to the peritoneal cavity; CXCL13 is also required for B1 cell homing to the omentum.^[^ ^48^ ^]^ In addition, after steady‐state migration to the intestinal lamina propria, peritoneal B1 cells secrete IgA natural antibodies to provide for immune control of the intestinal microbiota.^[^ ^12^ ^]^ Interestingly, IgA class switching in peritoneal B1 cells, and consequently B1 cell‐mediated intestinal IgA secretion, is controlled by retinoic acid/GATA6‐dependent TGF‐*β* production by LPMs.^[^ ^19^ ^]^ In line with this observation, retinoic acid and TGF‐*β* ad a synergistic effect on IgA class switch in peritoneal B1 cells in vitro.^[^ ^50^ ^]^

An additional function of LPMs linked to peritoneal homeostasis is surveillance for the detection of sterile peritoneal injury that, unless quickly repaired, could lead to the formation of peritoneal adhesions, that can turn into severe peritoneal disease, including intestinal occlusion and infertility in women.^[^ ^10^ ^]^ Surveillance for the detection of peritoneal damage or infection is essentially fulfilled by LPMs, and requires active patrolling of the peritoneal surface. In this regard, in a recent report, in which imaging of the peritoneal cavity, through the intact abdominal wall, was performed by intravital microscopy, LPMs were shown to move passively in a respiration‐dependent and random manner in the steady state, with speeds of up to 800 µm s^−1^.^[^ ^4^ ^]^ The role of LPMs in peritoneal tissue repair and adhesion formation is discussed in the next section.

## 6 Role of Large Peritoneal Macrophages in Repair of Peritoneal Injury

Damage of the abdominal or visceral peritoneum can be caused by sterile injury resulting from accidental trauma or abdominal surgery, or can result from peritoneal pathologies, such as infection, liver or intestinal diseases, or metastatic tumor growth. Recent studies, discussed below, have shed light on the mechanisms involved in repair of peritoneal sterile injury, and on the role of LPMs in this process.^[^ ^10^ ^]^ In contrast, how the peritoneum damaged by peritoneal infection or tumor metastasis is subsequently restored, remains to be explored in depth.

Sensing of damage in the peritoneal lining is achieved through the recognition of danger‐associated molecular patterns (DAMPs), released by damaged cells, including mesothelial cells, cells located in the submesothelial connective tissue, and potentially those forming the underlying tissues. DAMPs include constitutively‐expressed DAMPs, such as nuclear and mitochondrial DNA, nuclear and mitochondrial proteins (HMGB1, histones, cytochrome c), ATP, K^+^ ions, or S100 calcium binding proteins, inducible DAMPs, such as heat shock proteins, defensins, galectins, and IL‐1*α*, and extracellular DAMPs, such as hyaluronan or heparan sulfate.^[^ ^51^ ^]^ DAMP‐activated mesothelial cells trigger peritoneal inflammation through the release of proinflammatory cytokines and chemokines, that promote leukocyte recruitment to the damaged areas and complement activation, resulting in additional inflammation. This inflammatory reaction triggers tissue factor‐dependent fibrin polymerization as a result of an imbalance between fibrinogenesis and fibrinolysis, leading to the formation of a fibrin matrix, serving as the scaffold for wound repair. The latter involves the recruitment to the submesothelial compartment of leukocytes fulfilling a repair function, including LPMs, neutrophils, monocytes, and monocyte‐derived macrophages, recruitment of mesenchymal precursors, deposition of extracellular matrix, ingrowth of nerves and blood vessels, and remesothelialization of the damaged peritoneum.^[^ ^10^ ^]^ Persistence of peritoneal inflammation can lead to excessive fibrin deposition and, ultimately, to the formation of fibrous bridges between opposing peritoneal surfaces, containing nerves and blood vessels, called abdominal adhesions.^[^ ^52^ ^]^ Adhesions predominantly result from abdominal surgery, but can also be caused by infection, endometriosis, radiotherapy or peritoneal dialysis, and are associated with considerable morbidity that can involve life‐threatening complications.^[^ ^52^ ^]^

The analysis by electron microscopy of peritoneal healing after experimental surgical injury, revealing that macrophages adhered to the damaged tissue 24 h after injury was caused, and subsequently migrated into the wound,^[^ ^53^ ^]^ provided the first evidence on the possible role of macrophages in peritoneal injury repair. This hypothesis was further supported by intravital microscopy studies from Dr. P. Kubes' lab demonstrating that, after laser‐induced injury of the liver capsule, F4/80^high^ GATA6^+^ LPMs were recruited to the damaged areas, and migrated across the mesothelium into the liver injuries, within 1 h after laser‐induced injury.^[^ ^30^ ^]^ LPMs sensed damaged tissue through the recognition of ATP released by liver necrotic cells through the DAMP receptor PX27, and infiltrated the liver parenchyma through CD44‐mediating binding to hyaluronan present in the damaged areas. Interestingly, recruitment to the injured tissue triggered LPM proliferation and upregulation of molecules associated with an alternative activated /repair phenotype, such as CD206, CD273, and arginase 1. Accordingly, recruited LPMs actively contributed to necrotic cell removal, that was claimed to be critical for revascularization and tissue repair, as supported by experiments showing that healing of injured areas was delayed in clodronate‐loaded liposome‐mediated LPM‐depleted or GATA6‐deficient mice.^[^ ^30^ ^]^ A similar ATP‐induced recruitment, and CD44‐dependent migration to damaged area of F4/80^high^ GATA6^+^ LPMs was described in a model of intestinal thermal injury.^[^ ^54^ ^]^ Clodronate‐loaded liposome‐mediated LPM depletion experiments also supported the concept that LPMs contributed to injured intestinal repair in this experimental setting. However, whether, as described in this report, LPMs are recruited to the intestinal serosa after damage of the intestinal epithelium, would require further investigation, since it remains possible that this phenomenon was artefactual if, in these experiments, damage was not just limited to the intestinal luminal surface, but affected the intestinal mucosa and submucosa, taking into account the experimental strategy employed to address this issue in this study. On the other hand, regarding the experiments of LPM depletion by treatment with clodronate‐loaded liposomes, carried out to address the role of LPMs in liver or intestinal serosal repair,^[^ ^30^, ^54^ ^]^ whether the delayed wound healing observed in clodronate‐treated mice was due, at least in part, to the depletion of peritoneal monocyte‐derived macrophages and tissue‐resident macrophage populations present in the omentum, peritoneal membrane, or liver capsule, cannot be excluded. Indeed, monocytes have been demonstrated to be recruited to peritoneal injured areas, where they differentiate into monocyte‐derived macrophages that can promote tissue repair.^[^ ^55^ ^]^

In line with these observations, the concept that after LPMs attach to damaged mesothelium, they migrate into serosal injuries and fulfill a critical repair function, has been challenged by a recent report in which genetic fate‐mapping allowed to trace resident LPMs after liver sterile injury.^[^ ^56^ ^]^ These studies revealed that GATA6^+^ resident LPMs accumulated on the injured surface of the liver, but minimally invaded the necrotic liver parenchyma. Moreover, by using the diphtheria toxin dependent G6Mø‐CreER; R26‐tdTomato/iDTR mouse line, which allowed the genetic ablation of most GATA6^+^ resident LPMs, the authors concluded that the absence of GATA6^+^ resident LPMs did not significantly impact on liver wound healing, and thus that GATA6^+^ resident LPMs were not critical for damaged serosal tissue regeneration. Therefore, additional research has to be conducted to establish whether, and eventually how, LPMs contribute to peritoneal healing.

Interestingly, a recent report by Dr. P. Kubes' lab, based on the imaging of the peritoneal cavity after laser‐induced focal thermal peritoneal injury, by intravital microscopy through the intact abdominal wall, supports a direct role of LPMs in serosal repair.^[^ ^4^ ^]^ Indeed, resident GATA6^+^ LPMs were the first cells recruited to mesothelial injuries, a process that required peritoneal fluid shear flow. Recruited LPMs attached to the damaged peritoneum and completely covered the lesions 15 min after injury was caused, forming thrombus‐like structures, in a process that mirrored platelet aggregation in response to blood vessel injury. LPM aggregation was not dependent on canonical adhesion molecules or fibrin polymerization, but on scavenger receptors containing scavenger receptor cysteine‐rich (SRCR) domains, such as MARCO or MSR1, that bind to a high number of polyanionic ligands, and that are highly conserved throughout evolution from invertebrates. Indeed, in echinoderms, as the sea urchin, injury in the coelomic cavity led to the aggregation of coelomocytes, expressing SRCR‐containing homologs, that sealed the damaged areas.^[^ ^57^, ^58^ ^]^ LPMs were claimed to contribute to the repair of focal peritoneal lesions, by achieving a physical sealing of peritoneal injuries, since blockade of macrophage aggregation led to a delayed healing of injured parietal peritoneum.^[^ ^4^ ^]^

In contrast, using an experimental model of peritoneal adhesion formation induced by surgical sterile injury, involving the formation of a peritoneal button by suturing a portion of the peritoneal wall, high numbers of LPMs were shown to be recruited to the buttons within 3 h after surgery.^[^ ^4^ ^]^ In this iatrogenic setting, macrophages formed extensive aggregates that promoted the deposition of fibrin and the growth of scar tissue, leading to the formation of peritoneal adhesions within 7 days after surgery. Interestingly, the number and development of peritoneal adhesions was markedly reduced in mice in which LPMs were depleted by 24 h before surgery, supporting that LPMs contributed to peritoneal adhesion formation. Interestingly, by using a similar model of experimental adhesion formation, LPMs were shown to form a cell barrier over the fibrin clots formed in damaged mesothelial areas, a process leading to adhesion formation if the macrophage barrier was insufficient to cover the fibrin clot, but that precluded adhesion formation if the macrophage barrier completely shielded the fibrin clots.^[^ ^59^ ^]^ Indeed, IL‐4‐mediated reinforcement of macrophage barrier prevented adhesion formation, and could be the basis for the development of innovative treatments to prevent post‐operative adhesions. Therefore, although initial macrophage recruitment and aggregation, together with fibrin deposition, appears to be required for a correct serosal repair, it can also cause pathogenic scarring leading to adhesion formation, a situation that has been correlated with a low mesothelial fibrinolytic activity.^[^ ^52^ ^]^

**Figure 3:**
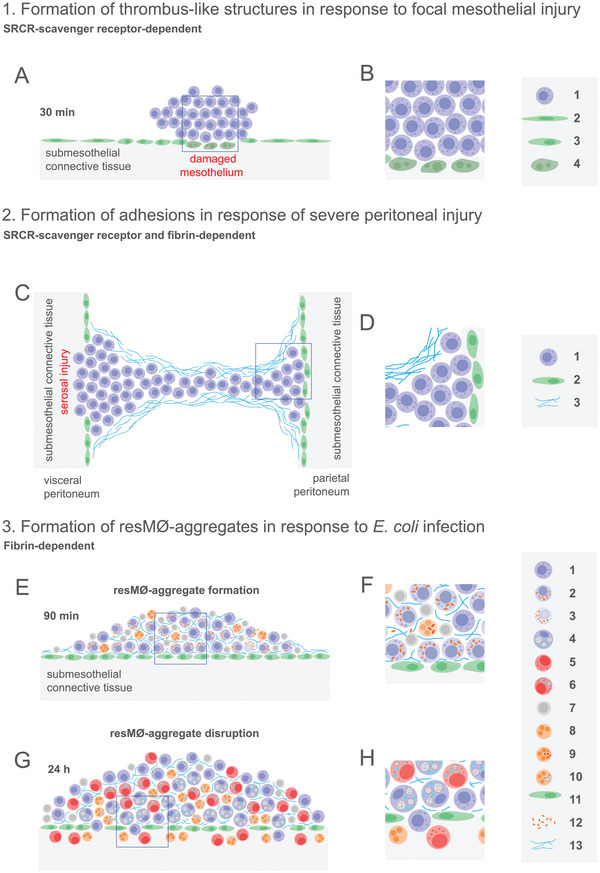
Recent experimental advances revealing the formation of mesothelium‐bound LPM aggregates induced by peritoneal injury or bacterial infection. 1) Formation of thrombus‐like structures in response to focal mesothelial injury. Imaging of the peritoneal cavity after laser‐induced focal thermal peritoneal injury, by intravital microscopy through the intact abdominal wall, revealed that LPMs attached to the damaged peritoneum, forming thrombus‐like structures dependent on scavenger receptors containing SRCR domains, that contribute to the repair of peritoneal lesions.^[^ ^4^ ^]^ A) Schematic representation of a thrombus‐like structure formed by LPM aggregates at 30 min after induction of injury. B) Enlargement of the area marked in (A). 1: LPMs; 2: mesothelial cells; 3: activated mesothelial cells; 4: apoptotic mesothelial cells. Reproduced with permission.^[^ ^4^ ^]^ Copyright 2021, AAAS. 2) Formation of adhesions in response of severe peritoneal injury. Using an experimental model of peritoneal adhesion formation induced by surgical sterile injury, high numbers of LPMs were reported to form extensive aggregates that promoted the deposition of fibrin and the growth of scar tissue, leading to the formation of peritoneal adhesions within 7 days after surgery.^[^ ^4^, ^59^ ^]^ C) Schematic representation of the induction of a peritoneal adhesion by extensive aggregation of LPMs over the injured tissue, forming a bridge between opposing peritoneal surfaces. D) Enlargement of the area marked in (C). 1: LPMs; 2: activated mesothelial cells; 3: fibrin. Reproduced with permission.^[^ ^4^ ^]^ Copyright 2021, AAAS. 3) Formation of resMØ‐aggregates in response to *E. coli* infection. Using a mouse model of sublethal sepsis, LPMs were recently demonstrated to promote the formation of mesothelium‐bound, fibrin‐dependent, resMØ‐aggregates, composed by sequentially‐recruited LPMs, B1‐cells, neutrophils, and moCs, that provided a physical scaffold allowing the interaction and function of peritoneal immune cells. During the resolution of infection, LPMs controlled the recruitment of moCs to resMØ‐aggregates, that were essential for fibrinolysis‐mediated resMØ‐aggregate disaggregation, leading to dampening of inflammation.^[^ ^47^ ^]^ E) Schematic representation of the formation of a resMØ‐aggregate at 90 min after infection. F) Enlargement of the area marked in (E). G) Schematic representation of the disruption a resMØ‐aggregate at 24 h after infection. H) Enlargement of the area marked in G). 1: LPMs; 2: LPMs containing bacteria; 3: necrotic LPMs containing bacteria; 4: LPMs containing dead cells and fibrin; 5: moCs; 6: moCs containing dead cells and fibrin; 7: B cells; 8: neutrophils; 9: neutrophils containing bacteria; 10: neutrophils containing dead cells; 11: activated mesothelial cells; 12: bacteria; 13: fibrin. Reproduced with permission.^[^ ^47^ ^]^ Copyright 2021, Elsevier.

In conclusion, the role of LPMs in sterile peritoneal damage repair is to a large extent dictated by the severity of the injury. LPMs promote adhesion formation after large peritoneal injury, but fulfill an essential function of rapid repair of focal mesothelial injuries, reminiscent of primitive repair mechanisms conserved throughout evolution (**Figure** **3**). On the other hand, the potential of LPMs to invade deep damaged submesothelial tissue, and contribute to its restoration together with monocyte‐derived macrophages and neutrophils, is still controversial and thus requires to be further investigated. In this regard, whether, as described for other macrophage populations, LPMs produce pro‐wound healing mediators, such as platelet‐derived growth factor, insulin‐like growth factor 1, TGF‐*β*1, or VEGF‐*α*,^[^ ^60^ ^]^ remains to be explored.

## 7 Role of Large Peritoneal Macrophages in Defense against Infection

The LPM transcriptional program in the steady state, dictated by the peritoneal microenvironment and driven by the GATA6 transcription factor, enables LPMs to carry out their homeostatic functions, but can be modulated by extracellular signals associated with pathological conditions, in order to allow LPMs to acquire an alternatively‐activated /repair phenotype in response to peritoneal injury or helminth invasion, or to perform immunological defense functions against microbial infections. Experimental evidence supports that LPMs fulfill a primary function of phagocytosis of invading microbial pathogens, reminiscent of the primitive phagocytic activity of coelomocytes in invertebrates, but few reports have actually contributed to define the role of LPMs in defense against peritoneal infection.

Depletion of LPMs by intraperitoneal injection of clodronate‐loaded liposomes resulted in a higher bacterial load and/or lower survival in different models of bacterial infection, including *Acinetobacter baumannii*, *Escherichia coli*, and *Enterococcus faecium*, supporting that LPMs contribute to control infection.^[^ ^5^, ^47^, ^61^, ^62^, ^63^ ^]^ Indeed, LPMs have been demonstrated to phagocytose bacteria in vivo,^[^ ^26^, ^47^, ^61^, ^64^ ^]^ but uptake of bacteria by LPMs can be nevertheless detrimental, as demonstrated in a mouse model of *Staphylococcus aureus* infection, in which bacteria escaped liver Kupffer cells, invaded the peritoneal cavity and proliferated inside LPMs, that were not capable of killing the pathogen, leading to cell lysis and infection spreading to the kidneys and visceral fat.^[^ ^65^ ^]^ Bacterial uptake by LPMs was reported to be negatively‐controlled by IL‐4 produced by peritoneal mast cells in response to *E. coli* infection, and in a mouse model of severe septic peritonitis, in which conditional ablation of mast cells led to increased survival.^[^ ^64^ ^]^ In this regard, the response of LPMs to peritoneal sepsis, remains poorly understood and have been essentially investigated in mouse models of sterile inflammation, induced by thioglycolate, zymosan or LPS. Under these conditions, LPMs undergo the so‐called macrophage disappearance reaction (MDR), a situation in which LPMs are not retrievable by peritoneal lavage at the early phase of the inflammatory reaction, and that appears to reflect the combination of LPM adherence to the mesothelium and LPM death.^[^ ^11^, ^66^ ^]^ The extent of the MDR and, correspondingly, the need LPM repopulation as inflammation subsides, are dependent on the strength of the inflammatory reaction, the re‐establishment of the LPM population occurring by CSF‐1‐mediated proliferation of the remaining LPMs.^[^ ^45^ ^]^ Two recent reports support that the MDR indeed reflects the response of LPMs to peritoneal injury, that involves the aggregation of LPMs to mesothelial damaged areas that physically seal focal peritoneal injuries^[^ ^4^ ^]^ or to peritoneal bacterial infection, involving the formation of fibrin‐dependent LPM aggregates bound to the mesothelium, required for efficient control of infection, as described below.^[^ ^47^ ^]^ An integrated view of recent experimental advances revealing the formation of mesothelium‐bound LPM aggregates induced by peritoneal injury or bacterial infection is summarized in Figure 3. Alternatively, during zymosan‐induced sterile inflammation, the MDR has been correlated to the formation of cellular clots free in the peritoneal cavity, that were essentially composed of LPMs and neutrophils, and were dependent on coagulation factor V expression by LPMs; similar clots were claimed to be formed in response to *E. coli* infection and to be required for efficient control of infection.^[^ ^5^ ^]^ In contrast, peritoneal helminth infections do not trigger MDR, but the accumulation of LPMs in the peritoneal cavity, through local IL‐4‐mediated proliferation.^[^ ^67^, ^68^ ^]^ Moreover, LPMs were reported to acquire an alternative (M2) activation phenotype during helminth infection, depending on fatty acid oxidation driven by CD36‐mediated uptake of triacylglycerol substrates and subsequent lysosomal lipolysis, a process needed for LPM proliferation and induction of protective responses against the parasite.^[^ ^69^ ^]^

During septic peritonitis, LPMs contribute to peritoneal inflammation by increasing vascular permeability, promoting neutrophil recruitment to the peritoneal cavity, and releasing pro‐inflammatory molecules, including nitric oxide, chemokines, and cytokines.^[^ ^11^ ^]^ These processes have to be finely regulated to ensure clearance of the pathogens while preventing acute inflammation and damage. In this regard, the regulation of the production by LPMs of IL‐1*β*, an extremely potent pro‐inflammatory molecule, has been uncovered in a recent report by Dr. P. Taylor's lab, showing that GATA6 negatively controls IL‐1*β* processing and release, in response to LPS stimulation, through prostaglandin I2‐dependent induction of IL‐10.^[^ ^70^ ^]^

Using a mouse model of sublethal sepsis, induced by intraperitoneal injection of the *E. coli* M6L4 strain isolated from the mouse intestine,^[^ ^71^ ^]^ LPMs have been recently demonstrated to play a pivotal role in defense against peritoneal bacterial infection.^[^ ^47^ ^]^ LPMs achieved an extremely efficient bacterial uptake, and promoted the formation of mesothelium‐bound multilayered structures, termed resident macrophage (resMØ)‐aggregates, that provided a physical scaffold allowing the interaction and function of peritoneal immune cells, and were therefore crucial for an efficient control of infection. resMØ‐aggregates were essentially composed by sequentially‐recruited LPMs, B1‐cells, neutrophils, and monocyte‐derived cells (moCs), and their formation was dependent on fibrin polymerization leading to a fibrin network^[^ ^47^ ^]^ (Figure 3). resMØ‐aggregates preferentially attached to the mesothelium covering the peritoneal wall, omentum, mesentery, and gonadal fat; in addition, LPMs migrated to the omental milky spots and FALCs present in the mesentery and gonadal fat. During the early phase of infection, *E. coli* infection led to death by pyroptosis of a significant proportion of LPMs, contributing to peritoneal inflammation. During the resolution of infection, LPMs controlled the recruitment of moCs to resMØ‐aggregates, that were essential for fibrinolysis‐mediated resMØ‐aggregate disaggregation, leading to dampening of inflammation.^[^ ^47^ ^]^

resMØ‐aggregate‐like structures may be required for antimicrobial immunity in other body cavities, such as the pleural cavity or the brain ventricular system, harboring macrophages in a fluidic environment, that may need to attach to the epithelium lining these cavities to fulfill their immune defense functions.

## 8 Role of Large Peritoneal Macrophages in Peritoneal Tumor Metastasis

Peritoneal tumor metastasis occurs after detachment of tumor cells from primary tumors exposed to the peritoneal cavity, being ovarian, gastric, colorectal, pancreatic, and appendicular cancers the most prone to peritoneal metastasis.^[^ ^72^, ^73^ ^]^ Patients with peritoneal metastasis have a poor prognosis and suffer from excruciating symptoms like intestinal obstruction, accumulation of malignant ascites, and severe incurable pain syndromes that severely compromise their quality of life in the terminal stages of disease. Peritoneal metastasis requires an initial interaction of detached tumor cells with the mesothelial lining that covers the peritoneal wall and the organs located in the peritoneal cavity.^[^ ^74^ ^]^ This initial step is followed by the invasion of the submesothelial space by tumor cells, leading to the remodeling of the peritoneal stroma, promoting the adhesion of tumor cells to the extracellular matrix, that supports their proliferation and thus, progression of metastasis.^[^ ^72^ ^]^ During peritoneal tumor metastasis, tumor cells display a preferential tropism to the omentum,^[^ ^75^ ^]^ that has been correlated to the hypoxic status of omental milky spots, possessing a unique vascular system that favors VEGF‐mediated angiogenesis,^[^ ^76^ ^]^ and to the production of fatty‐acid binding proteins, that promote lipid transfer from adipocytes to tumor cells, enhancing *β*‐oxidation metabolism and consequently tumor cell proliferation.^[^ ^77^ ^]^

LPMs are the first potential line of defense against peritoneal metastasis, yet recent experimental evidence supports that tumors can subvert peritoneal macrophage metabolism and/or function, leading to the acquisition of a pro‐tumor phenotype. Although tumor promoting functions of macrophages were initially associated to monocyte‐derived macrophages,^[^ ^78^ ^]^ embryonic tissue‐resident macrophages were recently demonstrated to be crucial role for tumor progression in mouse models of glioblastoma,^[^ ^79^ ^]^ pancreatic ductal adenocarcinoma,^[^ ^80^ ^]^ colon adenoma,^[^ ^81^ ^]^ and lung carcinoma.^[^ ^82^ ^]^ Peritoneal macrophages have been reported to promote metastatic tumor growth in mouse models of peritoneal cancer metastasis,^[^ ^34^, ^83^, ^84^, ^85^, ^86^, ^87^ ^]^ yet, as discussed below, the conclusions drawn from these studies are particularly divergent regarding the specific contribution of LPMs to peritoneal metastasis and the mechanistic basis of their tumor‐promoting role remains controversial (**Table** **1**).

**Table 1:** Experimental evidence of the tumor promoting function of peritoneal macrophages

Reference and Experimental model	Phenotypic and functional profile of tumor‐promoting macrophages	Mechanistic basis of the tumor‐promoting function of macrophages	Demonstration of the protumoral function of macrophages
Weiss et al, 2018 ‐ Intraperitoneal injection of ID8 ovarian tumor cells or B16 melanoma tumor cells.	‐ LPMs characterized in tumor‐bearing mice as F4/80^high^ GATA6^+^ cells that proliferated during tumor progression. ‐ No expression of prototypical protumoral genes (*IL10, TGFB and Retnla)*, but of proinflammatory genes (*IL6, TNFA* and *IL1B)*.	‐ Irg‐1/itaconate‐induced ROS production by LPMs promoted tumor progression through MAPK activation in tumor cells.	‐ LPM depletion by administration of clodronate‐loaded liposomes led to delayed tumor progression. ‐ Irg‐1 silencing in peritoneal macrophages by lentiviral shRNA led to delayed tumor progression.
Xia et al, 2020 ‐ Intraperitoneal injection of ID8 ovarian tumor cells.	‐ During tumor progression LPMs proliferated actively and generated Tim4^+^ TAMs. ‐ Tim4^+^ TAMs produced high levels of mitochondrial ROS production, and developed a high resistance to oxidative stress, through increased mitophagy.	‐ Not addressed.	‐ Autophagy deficiency in myeloid cells induced apoptosis in Tim4^+^ TAMs, due to accumulation of mitochondrial ROS, and led to delayed tumor progression.
Chow et al, 2021 ‐ Intraperitoneal injection of MC38 colon carcinoma cells.	‐ Not addressed.	‐ Tim4‐mediated sequestration of phosphatidylserine‐expressing anti‐tumor cytotoxic CD8^+^ T cells, impairing anti‐tumor immunity.	‐ Antibody‐mediated Tim4 blockade, or Tim4 deficiency, enhanced the efficacy of anti‐PD‐1 immunotherapy.
Goossens et al, 2019 ‐ Intraperitoneal injection of ID8 ovarian tumor cells.	‐ Monocyte‐derived F4/80^int^ MHCII^int^ macrophages gradually replaced LPMs. ‐ Expression of genes related to cholesterol metabolism and reverse cholesterol efflux. ‐ Macrophage reprogramming associated to a tumor promoting phenotype, involving increased arginine metabolism, and inhibition of IFN*γ*‐induced gene expression.	‐ IL‐4‐mediated, STAT6/PI3K‐dependent, macrophage reprogramming, triggered by cholesterol efflux, led to increased arginine metabolism, that promoted immunosuppression and tumor growth.	‐ Blocking IL‐4 signaling by anti‐IL‐4R delayed tumor growth. ‐ Prevention of cholesterol efflux by genetic deletion of ABC membrane cholesterol efflux transporters delayed tumor growth.
Etzerodt et al, 2020 ‐ Intraperitoneal injection of ID8 ovarian tumor cells.	‐ Lyve‐1^+^ CD163^+^ Tim4^+^ tissue‐resident omental macrophages that expressed genes associated with positive regulation of JAK‐STAT signaling, linked with macrophage self‐renewal and genes associated with tumor‐promoting functions. ‐ Lyve‐1^+^ CD163^+^ Tim4^‐^ monocyte‐derived omental macrophages expressed genes associated with tumor‐promoting functions.	‐ CD163^+^ Tim4^+^ macrophages promoted the acquisition of cancer stem cell and epithelial‐to‐mesenchymal transition characteristics by ovarian cancer cells.	‐ Depletion of CD163^+^ Tim4^+^ macrophages by diphtheria toxin‐mediated specific ablation prevented metastatic spread and invasive disease. ‐ Depletion of CD163^+^ macrophages by CD163‐targeted, doxorubicin‐loaded, lipid nanoparticles, delayed omental tumor progression.
Zhang et al, 2021 ‐ Intraperitoneal injection of ID8 ovarian tumor cells.	‐ Lyve‐1^high^ MHCII^low^ mesenteric and peritoneal wall macrophages displaying an alternatively‐activated expression profile.	‐ Not addressed.	‐ Genetic ablation of Lyve‐1^high^ macrophages and omentectomy delayed tumor progression within ascites.
Yin et al, 2016 ‐ Intraperitoneal injection of ID8 ovarian tumor cells.	‐ During tumor progression peritoneal cavity macrophages proliferated and switched gradually from expressing M1 (*Ccr2, Ifnar, iNOS*) to M2 genes (*Cd206, arginase 1, Cd163*). ‐ Whether tumor‐associated macrophages resulted from proliferation of resident LPMs, and/or differentiated from monocytes, was not addressed.	‐ Formation of spheroids composed of macrophages and tumor cells through *β*2 integrin‐ICAM‐1 interactions. ‐ Production of EGF by macrophages promoted tumor growth by EFGR signaling in tumor cells, triggering VEGF release by tumor cells and autocrine VEGFR signaling.	‐ Blockage of spheroid formation by clodronate‐liposome‐mediated macrophage depletion or anti‐ICAM‐1 antibodies delayed tumor growth. ‐ Pharmacological blockade of EGFR delayed tumor growth.

LPMs were reported to infiltrate primary ovarian tumors, in an orthotopic syngeneic ovarian cancer model, in which Upk10 ovarian tumor cells were injected in the ovarian bursa. The number of tumor‐infiltrating LPMs was markedly reduced in mice deficient in RXR*αβ* in myeloid cells, displaying a disrupted LPM homeostasis, and this was associated with a delayed tumor growth, suggesting that LPMs promoted tumor progression, yet the mechanistic basis of this pro‐tumor effect was not addressed.^[^ ^34^ ^]^ Interestingly, ovarian and melanoma peritoneal tumors were reported to subvert peritoneal macrophage metabolism, resulting in Irg1‐induced production of itaconic acid by LPMs from tumor‐bearing mice, that led to a fatty acid oxidation‐mediated increase in oxidative phosphorylation and glycolysis.^[^ ^86^ ^]^ This caused an enhanced mitochondrial ROS production by LPMs, that promoted tumor progression, through ROS‐mediated MAPK activation in tumor cells. Tumor growth was reduced after peritoneal macrophage depletion by administration of clodronate‐loaded liposomes, or Irg‐1 silencing in peritoneal macrophages by lentiviral shRNA leading to decreased ROS production, supporting that LPMs had a tumor‐promoting function.^[^ ^86^ ^]^ Interestingly, in this experimental setting, the protumor phenotype of LPMs was not related to the expression of prototypical protumor genes, such as *IL10*, *TGFB*, and *Retnla*, but proinflammatory genes, such as *IL6*, *TNFA*, and *IL1B*. In line with this report, during ovarian peritoneal metastasis, LPMs were demonstrated to proliferate and generate a population of Tim4^+^ tumor‐associated macrophages (TAMs), that displayed increased mitochondrial activity and mitochondria‐related ROS production, and developed a high resistance to oxidative stress through increased mitophagy, that led to the elimination of damaged mitochondria.^[^ ^88^ ^]^ Interestingly, mitophagic activity was claimed to correlate with a high arginase‐1 activity increasing arginine metabolism in Tim4^+^ TAMs, resulting in low levels of arginine, that caused mTORC1‐mediated mitophagy inhibition.^[^ ^88^ ^]^ Mice in which myeloid cells were deficient in FIP200, a protein essential for the induction of autophagy,^[^ ^89^ ^]^ displayed a significant reduction in Tim4^+^ TAMs, due to apoptosis caused by accumulation of damaged mitochondria and mitochondrial ROS, and a delayed peritoneal ovarian tumor progression, supporting that Tim4^+^ TAMs promoted tumor progression.^[^ ^88^ ^]^ An alternative tumor‐promoting function of LPMs has been reported in a recent study, based on a model of colon carcinoma peritoneal metastasis, claiming that LPMs supported tumor progression by impairing anti‐tumor CD8^+^ T cell immunity, through Tim4‐mediated sequestration of phosphatidylserine‐expressing anti‐tumor cytotoxic CD8^+^ T cells, preventing their proliferation.^[^ ^85^ ^]^ Indeed, antibody‐mediated blockade of Tim4, or Tim4 deficiency, enhanced the efficacy of anti‐PD‐1 immunotherapy, that was associated with an increase in CD8^+^ T cells in the peritoneal cavity. However, an in vivo imaging of Tim4‐mediated macrophage‐CD8^+^ T cell interactions, demonstrating the sequestration hypothesis, was not provided in this report.

A recent study from Dr. T. Lawrence's lab has demonstrated, in a peritoneal ovarian cancer model, that LPMs were progressively replaced by monocyte‐derived macrophages, that gradually upregulated genes related to cholesterol metabolism and reverse cholesterol efflux.^[^ ^87^ ^]^ Indeed, tumor cells promoted cholesterol efflux in monocyte‐derived macrophages that drove IL‐4‐mediated, STAT6/PI3K‐dependent, macrophage reprogramming, involving increased arginine metabolism, and inhibition of IFN*γ*‐induced gene expression, that promoted tumor progression. Genetic deletion of ABC membrane cholesterol efflux transporters prevented cholesterol efflux and reverted the tumor promoting functions in peritoneal monocyte‐derived macrophages.^[^ ^87^ ^]^ Whether these monocyte‐derived macrophages acquired, in the long‐term a resident LPM identity was not addressed in this study. In a second report from the same group, based on the same peritoneal metastasis model, two populations of Lyve‐1^+^ omental macrophages, with different protumor functions, were described. Lyve‐1^+^ CD163^+^ Tim4^+^ tissue‐resident embryonic‐derived macrophages were required for metastatic spread of ovarian cancer cells, whereas both Lyve‐1^+^ CD163^+^ Tim4^+^, and Lyve‐1^+^ CD163^+^ Tim4^−^ monocyte‐derived omental macrophages, contributed to tumor progression in the omentum.^[^ ^83^ ^]^ Transcriptomic analyses revealed that CD163^+^ Tim4^+^ macrophages expressed genes associated with positive regulation of JAK‐STAT signaling, linked with macrophage self‐renewal,^[^ ^90^ ^]^ while both CD163^+^ Tim4^+^ and CD163^+^ Tim4^−^ macrophages expressed genes associated with tumor‐promoting functions, such as angiogenesis, blood vessel development, and tissue remodeling.^[^ ^91^ ^]^ Specific depletion of CD163^+^ Tim4^+^ macrophages did not affect omentum metastasis seeding, but prevented the development of invasive disease, that correlated with the ability of CD163^+^ Tim4^+^ omental macrophages to promote the acquisition of cancer stem cell and epithelial‐to‐mesenchymal transition characteristics by ovarian cancer cells.^[^ ^83^ ^]^ The developmental and functional link between LPMs and omental CD163^+^ Tim4^+^ macrophages remains to be established. In line with these results, CCR1‐deficiency in ovarian tumor cells led to a reduction in their omentum seeding, that was claimed to depend on the expression of the CCR1‐ligand CCL6 by omental macrophages.^[^ ^92^ ^]^ Interestingly, G. Randolph's lab identified recently two populations of F4/80^high^ ICAM‐2^−^ CD206^+^ tissue‐resident macrophages, mainly located in the mesentery and peritoneal wall, characterized as Lyve‐1^low^ MHCII^high^ and Lyve‐1^high^ MHCII^low^ cells, that were not dependent on the GATA6 transcription factor.^[^ ^37^ ^]^ Lyve‐1^high^ MHCII^low^ macrophages were shown to be of embryonic origin, depend on CFS1 and display an alternatively‐activated macrophage phenotype and, based on their transcriptional profile, claimed to be related to omental, tumor‐promoting, Lyve‐1^+^ CD163^+^ Tim4^+^ macrophages, recently described.^[^ ^83^ ^]^ The potential protumor function of Lyve‐1^high^ macrophages was assessed in mice with genetic ablation of Live‐1^+^ cells, that were omentectomized, in order to exclude the tumor promoting function of omental Lyve‐1^+^ CD163^+^ Tim4^+^ macrophages. Ovarian peritoneal tumor growth was delayed in these mice, particularly within ascites, supporting that mesenteric and peritoneal wall Lyve‐1^high^ MHCII^low^ macrophages promoted tumor progression.^[^ ^37^ ^]^

Interestingly, in a mouse model of peritoneal ovarian cancer, based on the intraperitoneal injection of ID8 tumor cells, spheroids formed by peritoneal macrophages and tumor cells, through *β*2 integrin‐ICAM‐1 interactions, were detectable in the ascites from 3 weeks after ID8 injection.^[^ ^84^ ^]^ Macrophages present in the peritoneal cavity increased in number almost tenfold along the first 8 weeks after ID8 injection, and switched gradually from expressing M1 (*Ccr2*, *Ifnar*, *iNOS*) to M2 genes (*Cd206*, *arginase 1*, *Cd163*). Production of EGF by macrophages promoted tumor growth by EFGR signaling in tumor cells, triggering VEGF release by tumor cells and autocrine VEGFR signaling. Prevention of spheroid formation by macrophage depletion or anti‐ICAM‐1 antibodies, or pharmacological blockade of EGFR, significantly delayed tumor growth,^[^ ^84^ ^]^ supporting that peritoneal macrophages are critical for ovarian cancer metastatic progression by driving spheroid formation. Whether the TAM population involved in spheroid formation resulted from proliferation of resident LPMs, and/or differentiated of ii‐moLPMs, was not addressed in this report.

In conclusion, research developed over the last years has demonstrated that, during peritoneal cancer metastasis, LPMs contribute to tumor progression, most likely reflecting both an intrinsic protumor potential, and the acquisition of tumor‐promoting functions, through tumor‐induced changes in their metabolism. Different molecular mechanisms have been proposed to explain the protumor function of LPMs, summarized in Table 1, but additional experimental work is needed to integrate these data and achieve an in‐depth and comprehensive understanding of the role of LPMs in peritoneal tumor progression. Importantly, in addition to LPMs, different peritoneal macrophage subpopulations with protumor potential have been recently identified, including Lyve‐1^+^ CD163^+^ omental macrophages, and Lyve‐1^high^ MHCII^low^ mesenteric and peritoneal wall macrophages that, consequently, have to be taken into account in the design of experiments aiming at exploring the tumor promoting function of peritoneal macrophages, and in the development of immunotherapeutical antitumor strategies.

## 9 Concluding Remarks

Research developed over the last few years has significantly broadened our understanding of LPM biology by defining the dynamics of the replacement of resident embryonic LPMs by resident moLPMs, leading to sexually dimorphic phenotype and function, and explaining how LPMs, that move passively in the fluidic environment of the peritoneal cavity in the steady state, form mesothelium‐bound LPM aggregates to fulfill a crucial role in repairing peritoneal injuries and controlling microbial and parasitic infections.

On the other hand, a number of recent reports have demonstrated that, during peritoneal cancer metastasis, LPMs contribute to tumor progression, most likely reflecting an intrinsic protumor potential and/or the acquisition of tumor‐promoting functions, through tumor‐induced changes in their metabolism. Different molecular mechanisms have been proposed to explain the protumor function of LPMs, summarized in Table 1, but additional experimental work is needed to integrate these data and achieve an in‐depth and comprehensive understanding of the role of LPMs in peritoneal tumor progression. Importantly, in addition to LPMs, different peritoneal macrophage subpopulations with protumor potential have been recently identified, including Lyve‐1^+^ CD163^+^ omental macrophages, and Lyve‐1^high^ MHCII^low^ mesenteric and peritoneal wall macrophages that, consequently, have to be taken into account in the design of experiments aiming at exploring the tumor promoting function of peritoneal macrophages, and in the development of immunotherapeutical antitumor strategies. Importantly, these studies have revealed that the protumor potential of LPMs can be reverted by strategies blocking tumor‐induced subversion of LPM metabolism, providing the basis for the development of novel immunotherapeutic approaches against peritoneal tumor metastasis based on peritoneal macrophage reprogramming.

## Conflict of Interest

The authors declare no conflict of interest.
